# Effect of a Home Bleaching Gel Containing Chitosan and Theobromine on Tooth Surface Roughness, Microhardness, and Colour Change

**DOI:** 10.3390/gels11121014

**Published:** 2025-12-17

**Authors:** Safıya Temizyurek, Derya Gursel Surmelioglu

**Affiliations:** 1 Oral and Dental Health Center, Gaziantep 27410, Turkey; 2 Department of Restorative Dentistry, Faculty of Dentistry, Gaziantep University, Gaziantep 27410, Turkey

**Keywords:** theobromine, chitosan, microhardness, surface roughness, bleaching

## Abstract

This study aimed to evaluate the effect of experimental bleaching gels containing chitosan and theobromine and compare their performance in terms of tooth surface roughness, microhardness, and colour change with the bleaching gels BioWhiten ProHome and FGM Whiteness Perfect. One hundred and forty-four upper central incisors were used for microhardness, surface roughness, and colour change analyses (n = 12). Prior to bleaching, surface roughness was measured using a profilometer, microhardness was analysed using a Vickers hardness test, and colour was measured using a spectrophotometer. For Group 1, the treatment consisted of an experimental gel containing chitosan–theobromine (16% CP); for Group 2, it was an experimental gel containing chitosan–theobromine (6% HP); for Group 3, it consisted of BioWhiten ProHome (6% HP); and for Group 4, it consisted of FGM Whiteness Perfect (16% CP). Microhardness and surface roughness tests were performed under the same conditions before bleaching, after bleaching, and 14 days after the initial treatment. Colour analysis was performed before the bleaching, during the application, 24 h after bleaching, and at 7 and 14 days after treatment. *p* < 0.05 was considered significant. No statistically significant increase in microhardness values after bleaching was detected in any group (*p* > 0.05), effective bleaching was detected in all groups, and the highest efficacy was observed in Group 4 (*p* < 0.05). The experimental gels containing theobromine and chitosan resulted in effective bleaching and did not exert any negative effects regarding surface roughness or microhardness.

## 1. Introduction

Bleaching treatments are applied to vital teeth. These treatments can be in-office techniques, applied in the office by physicians; at-home techniques, performed by the patients themselves at home according to a physician’s guidance; and bleaching treatments performed without a physician’s intervention [1].

The peroxide ratios of gels used for at-home and in-office bleaching techniques vary. Gels containing peroxide at low concentrations are used at home with the help of specially prepared plates. In contrast, the in-office bleaching techniques involve applying high-concentration gels directly to the tooth surface [2]. Home bleaching treatments performed by patients using personalised plaques are cheaper and follow a simpler protocol than in-office bleaching applications. Moreover, the time spent in the clinic is shorter with in-office techniques [3]. While the high-concentration bleaching gels used for in-office bleaching cause more gingival irritation and tooth sensitivity, the bleaching activities of both techniques are similar [4], with both relying on gels that contain hydrogen peroxide (HP) or carbamide peroxide (CP) [5].

The high toxicity and carcinogenicity of the agents contained in the gels available on the market, as well as the large number of side effects, has led to the development of new materials. However, the limited number of clinical trials conducted on this subject indicates that this study fills a gap in the existing literature.

Studies found in the literature have evaluated many aspects of bleaching treatments, such as their application methods, agents, agent concentrations, efficacy, and sensitivity; however, the superiority of any method or agent over another remains unclear [6]. The type of agent, the duration of contact, and the activation devices used according to the selected protocol do not result in a clear difference in terms of effectiveness; however, they may differ in their respective impacts on tooth and gum sensitivity. These impacts may be related to the more correct and meticulous application of the bleaching method by a physician compared with that by a patient. The general consensus on tooth sensitivity is that the risk of sensitivity increases because the teeth are exposed to a higher concentration of HP and light activation in in-office bleaching treatments compared to at-home bleaching treatments [1]. Applying a higher concentration gel or increasing the contact time also increases sensitivity [7]. In terms of effectiveness, it is believed that one session of in-office bleaching treatment is equivalent to 2 weeks of at-home bleaching [4]. We preferred to formulate the experimental bleaching gel we prepared for use in our study as a home whitening product.

Chitosan is a bioactive, antimicrobial, biocompatible, non-toxic material. In addition to these properties, some studies suggest that chitosan prevents plaque formation and demineralisation in the mouth and increases osteogenesis and healing [8,9]. Chitosan possesses strong cationic and chelating properties, enabling it to bind electrostatically to negatively charged enamel surfaces and enhance surface adhesion [10]. This interaction has been shown to inhibit bacterial attachment, reduce biofilm formation, and modulate calcium–phosphate ion exchange, thereby limiting mineral loss under acidic conditions [11,12]. Additionally, chitosan plays a role in stabilising amorphous calcium phosphate (ACP) nanoclusters, supporting the nucleation and deposition of mineral phases on demineralized enamel surfaces, which contributes to biomimetic remineralisation [13]. For this reason, in the experimental gels used in our study, chitosan was used as a thickening or carrier agent for its bioadhesive and remineralising properties.

Theobromine is a water-insoluble, crystalline, methylxanthine alkaloid from the methylxanthine family, which is obtained from the cocoa plant [14]. Theobromine exhibits enamel-strengthening effects through distinct biochemical mechanisms. A study indicates that theobromine enhances hydroxyapatite crystallinity by promoting larger and more stable crystal structures, increasing enamel resistance to acid dissolution [15]. Many studies report that theobromine contributes to remineralisation, positively affects mineral changes in superficial enamel layers, increases surface microhardness, and reduces surface roughness [16,17,18,19]. Furthermore, theobromine can inhibit bacteria and increase saliva buffering capacity [16]. In addition, theobromine blocks dentin tubules by enlarging the structure of hydroxyapatite crystals and facilitating their precipitation, and it is also effective in the treatment of dentin sensitivity [15,20,21]. These mechanisms may play a complementary role in preserving enamel quality during oxidative bleaching procedures, where demineralisation and softening are potential concerns.

Although both agents have individually demonstrated beneficial effects, no comprehensive evaluation has previously assessed their combined incorporation into a bleaching gel and the resulting impact on enamel surface roughness, microhardness, and colour stability. This gap in the literature underscores the novelty of this study.

The current study investigates an experimental bleaching gel simultaneously enriched with chitosan and theobromine, a formulation concept not previously explored in enamel-related outcomes. Understanding how these bioactive components interact within a whitening system is clinically significant, given the rising demand for safer home-use bleaching products that maintain efficacy while offering protective benefits [22].

The findings of this study therefore contribute important preliminary evidence toward the development of bleaching formulations capable of reducing adverse effects on enamel while providing effective whitening outcomes.

Given the above, experimental bleaching gels containing chitosan and theobromine were developed to provide effective bleaching and minimise any side effects that may occur after bleaching applications; these gels were produced with low concentrations of HP and CP agents. To ensure standardisation when choosing the gels we applied, gels containing 6% HP, 16% CP, and an equivalent concentration of active agent were preferred.

In this study, we compared the newly developed experimental bleaching gels with the Bio-Whiten ProHome bleaching gel containing 6% HP (BIO) and the FGM Whiteness Perfect home-use bleaching gel containing 16% CP (FGM). We aimed to evaluate the effects of the experimental gels on tooth surface roughness, microhardness, and colour change.

Despite the protective and remineralizing effects of chitosan and theobromine when used individually, there is limited literature on their combined use in a bleaching gel formulation. Current whitening systems rely predominantly on peroxide-based agents, which effectively bleach tooth colour but may induce undesirable changes in enamel microhardness, surface roughness, and mineral integrity. Considering that chitosan provides bioadhesion and ion-modulating remineralization, while theobromine enhances hydroxyapatite crystallinity and acid resistance, the simultaneous incorporation of these agents has the potential to offer synergistic enamel protection during bleaching. However, no study to date has evaluated whether such a dual-component formulation can mitigate peroxide-related enamel alterations while maintaining whitening efficacy. This absence of scientific data represents a significant gap in the literature and highlights the clinical relevance of investigating a bleaching gel enriched with both chitosan and theobromine.

The null hypotheses were as follows:

(1) Theobromine, when added to bleaching gels, does not adversely affect dental surface roughness, microhardness, or colour change.

(2) Chitosan, when added to bleaching gels, does not adversely affect dental surface roughness, microhardness, or colour change.

## 2. Results and Discussion

### 2.1. Microhardness Findings

VHN_1_: Measured microhardness values before application

VHN_2_: Measured microhardness values after application

VHN_3_: Measured microhardness values 14 days after application

In the comparison between groups; There was no statistically significant difference among the VHN1, VHN2, and VHN3 values (*p* > 0.05).

Following the intra-group comparison, although there was a statistically insignificant increase in VHN_2_ values in all groups, the greatest increase was observed in ECP (Group 1). Although non-significant decreases in VHN_3_ values were observed in all groups, the greatest decrease was observed in EHP (Group 2) (*p* > 0.05).

VHN_1_, VHN_2_, and VHN_3_ with standard deviation (±sd) are presented in Table 1.

### 2.2. Surface Roughness Findings

Ra_1_: Values of surface roughness measured before application

Ra_2_: Values of surface roughness measured after application

Ra_3_: Values of surface roughness measured 14 days after application

In the comparison between groups, there were no significant differences in Ra_1_ values among the groups (*p* > 0.05). In addition, there were no significant differences between ECP (Group 1), EHP (Group 2), or FGM (Group 4) regarding Ra_2_ or Ra_3_ values (*p* > 0.05); however, significant differences were observed between these three groups and BIO (Group 3) (*p* < 0.05). In the comparisons within groups, although there were significant decreases in Ra_2_ values in ECP (Group 1), EHP (Group 2), and FGM (Group 4), the greatest decrease was observed in ECP (Group 1) (*p* < 0.05). In BIO (Group 3), a statistically insignificant increase in surface roughness was observed. A statistically insignificant decrease was observed in ECP (Group 1), EHP (Group 2), BIO (Group 3), and FGM (Group 4) (*p* > 0.05).

Ra_1_, Ra_2_ and Ra_3_ with standard deviation (±sd) are presented in Table 2.

### 2.3. Results of the Colour Difference Values (ΔE_Lab_)

ΔE_Lab1_: Colour change values measured before application and 7 days after the start of the application

ΔE_Lab2_: Colour change values measured before application and 24 h after the end of the application

ΔE_Lab3_: Colour change values measured before application and 7 days after the end of the application (day 21 end of the day)

ΔE_Lab4_: Colour change values measured before application and 14 days after the end of the application (day 28 end of the day)

The mean ΔE_Lab_ values (±sd) of all groups at all time points are presented in Table 3.

In the comparison between groups, there were no significant differences in ΔE_Lab_ values between any groups (*p* > 0.05). The largest ΔE_Lab1_ and ΔE_Lab2_ values were observed in FGM (Group 4), the smallest ΔE_Lab1_ value was observed in EHP (Group 2), and the smallest ΔE_Lab2_ value was observed in BIO (Group 3). In the intragroup comparison, there were significant differences between all ΔE_Lab1_ and ΔE_Lab2_ values in all groups; however, there were no statistically significant differences between ΔE_Lab2_, ΔE_Lab3,_ or ΔE_Lab4_ values. There were no significant differences between ΔE_Lab2_ and ΔE_Lab3_, ΔE_Lab2_ and ΔE_Lab4_, or ΔE_Lab3_ and ΔE_Lab4_ values in all groups.

ΔWI_D1_: Values before application and 7 days after the start of the application

ΔWI_D2_: Values before application and 24 h after the end of the application

ΔWI_D3_: Values before application and 7 days after the end of the application (day 21 end 260 of the day)

ΔWI_D4_: Values before application and 14 days after the end of the application (day 28 end of the day)

The mean ΔWI_D_ values (±sd) of all groups at all time points are presented in Table 4.

In the comparison between groups, there was no significant difference in ΔWI_D_ values between any groups (*p* > 0.05). In the intra-group comparison, while there was a significant difference between ΔWI_D1_ and ΔWI_D2_ values in all groups (*p* < 0.05), no statistically significant differences were observed between ΔWI_D2_, ΔWI_D3_, or ΔWI_D4_ values. There were no significant differences between ΔWI_D2_ and ΔWI_D3_, ΔWI_D2_ and ΔWI_D4_, or ΔWI_D3_ and ΔWI_D4_ values in any group.

A high level of positive correlation was found between the whiteness indexes calculated by CIE L*a*b* and ΔWI_D_ formulas and colour changes in all groups (*p* < 0.05, r > 0.6) (Table 5).

We therefore adopted the following hypotheses:

(1) Adding theobromine to bleaching gels does not adversely affect dental surface roughness, microhardness, or colour change.

(2) Adding chitosan to bleaching gels does not adversely affect dental surface roughness, microhardness, or colour change.

The concentrations of chitosan and theobromine used in this study were selected based on the previously published literature in which their antibacterial, anti-demineralization, and remineralizing effects on dental tissues were demonstrated. Studies on chitosan have shown that concentrations ranging from 0.1% to 2% can reduce enamel demineralisation and provide antibacterial activity [13,23,24]. Khamverdi et al. [23] reported that formulations containing chitosan reduced plaque accumulation, while Pini et al. [24] and Arnaud et al. [13] demonstrated that 1–2% chitosan promoted mineral gain on the enamel surface. Similarly, in vitro studies have shown that theobromine at concentrations between 0.1% and 2% can increase enamel microhardness and reduce acid solubility [15,25,26]. Premnath et al. [25] demonstrated that 0.1–0.2% theobromine increased hydroxyapatite crystal size, whereas and Amaechi et al. [15] reported that 1% theobromine provided a remineralisation effect comparable to fluoride. Therefore, the concentrations used in our study are consistent with previous research and represent scientifically appropriate levels for evaluating the potential therapeutic effects of these components.

### 2.4. Evaluation of Microhardness

In previous studies where surface roughness and microhardness measurements were evaluated after bleaching treatments, the different gel components used, the concentration of the agent, its pH, and the duration of application affected the surface roughness and microhardness values [26]. However, in these studies, the results are not compatible with each other.

Miranda et al. [27] conducted a study in which they used bleaching gels containing 10% CP and 10% HP to investigate the effects of different bleaching gel contents and application times on the enamel surface. Surface roughness analyses revealed that the gel containing 10% HP affected the surface roughness by disrupting the hydroxyapatite sequence in the enamel structure 4 weeks after the start of treatment, and the gel containing 10% CP affected the surface roughness 8 weeks after the start of treatment; however, it was emphasised that the increase in roughness was not sufficient for biofilm retention. In an in vitro study by Faraini-Romano et al. [28], the effects on surface roughness values of agents with CP contents of 10% and 22% and HP contents of 38%, 18%, and 7.5% were compared. In that study, no significant differences between the groups in terms of surface roughness values after the bleaching processes were evident. In contrast, in an in vitro study by Carvalho et al. [29], bleaching gels containing 20–45% CP and 9.5–38% HP were compared, and the greatest increase in surface roughness was observed following treatment with the gel containing 38% HP, while the lowest increase was observed following treatment with the gel containing 20% CP.

Mohammed et al. evaluated the effects of different remineralising gels on susceptibility to recolouration and surface roughness after bleaching treatment. Four different remineralising agents were applied to the enamel surface after a bleaching agent containing 10% CP was applied, and the results were compared with a control group. The group without a remineralised adhesive produced the highest surface roughness values, and it was concluded that the increase in surface roughness also increased susceptibility to recolouring [30].

Pini et al. conducted a study aiming to evaluate the effect on tooth properties and bleaching effectiveness of adding chitosan to a HP-containing bleaching gel. The authors stated that there was no significant difference between the group treated with HP + 2% chitosan gel and the control group. However, there was a significant increase in the surface roughness of the group treated with HP-containing gel [31]. Ozcetin et al. conducted a study comparing the effects on enamel surface roughness of an experimental 6% HP bleaching gel enriched with chitosan and bleaching gels containing 25% and 40% HP. They found no significant differences between the groups in terms of surface roughness. It was concluded that chitosan prevented an increase in surface roughness values without affecting the bleaching efficiency of the gel [32].

Wulandari et al. investigated whether tooth brushing with toothpaste containing theobromine prevented tooth discolouration caused by coffee consumption. It was determined that the acidic environment caused by chlorogenic acid and other types of acids in coffee reduces the crystal size and causes mineral loss on the hydroxyapatite surface. The remineralising effect of theobromine reduced this roughness and thus prevented discolouration [19]. According to the results obtained in the present study, the ECP (Group 1) and EHP (Group 2) gels reduced surface roughness values via the remineralising effect of theobromine and chitosan, while the FGM Whiteness Perfect home-use bleaching gel (Group 4) reduced the surface roughness values due to its sodium fluoride content. It is believed that the increase in surface roughness observed in the group treated with the BioWhiten ProHome home-use bleaching gel (Group 3) was due to the citric acid content of the gel. In a study in which they investigated the effect of over-the-counter teeth bleaching products on enamel surface roughness, Abidia et al. reported a significant increase in surface roughness in a group treated with 0.25% citric acid [33]. It was believed that the increase in tooth roughness was nullified by nanohydroxyapatite, a remineralising agent contained in the gel.

### 2.5. Evaluation of Surface Roughness

Mushashe, et al. conducted a study to evaluate the effects of a home-use bleaching agent containing 10% CP and an in-office bleaching agent containing 35% HP on enamel microhardness. They observed a decrease in microhardness values in both groups, but this decrease was not statistically significant [34]. Elfallah et al. conducted an in vitro study on bleaching gels to investigate the effects of their protein content and mechanical properties on tooth enamel. Bleaching agents containing 16% CP and 35% HP were compared, and although there was no significant difference between the two groups in terms of microhardness, the decrease in microhardness was significant in both groups [35]. Maia et al. evaluated the effect of home-use bleaching gels containing 10% CP and 7.5% HP on enamel microhardness and reported no significant difference between the groups. However, it has been reported that gels containing 7.5% HP tend to reduce enamel microhardness [36].

Attin et al. investigated how the addition of 0.5% fluoride to a home-use bleaching gel containing 10% CP affected enamel microhardness and the time it took for microhardness to return to baseline values. They found that gel containing fluoride caused significantly less loss of hardness than fluoride-free gel. It was concluded that the time required to return to baseline values was significantly shorter for the gel containing fluoride [37]. There was a decrease in microhardness values in the teeth that underwent bleaching treatment; however, this decrease could be reversed with the use of a solution containing 0.05% fluoride [38].

Suryana et al. aimed to compare the effects of toothpaste containing theobromine and hydroxyapatite on enamel microhardness after soaking tooth samples in a carbonated drink. The microhardness values in the theobromine group were found to be significantly higher than in the hydroxyapatite group [39].

In our study, microhardness values increased in all groups, and no significant differences were observed between VHN_1_, VHN_2_, and VHN_3_ values in the comparison between groups. However, in the intra-group comparison, there was a non-significant increase in microhardness values after bleaching in all groups, and the greatest increase was observed following treatment with ECP (Group 1). The increase in microhardness following ECP (Group 1) and EHP (Group 2) treatment was associated with chitosan and theobromine, which are natural remineralising agents added to the gels used in these groups. We believe that this effect was due to nanohydroxyapatite in BIO (Group 3) treated with the BioWhiten ProHome home-use bleaching gel and the sodium fluoride in FGM (Group 4) treated with the FGM Whiteness Perfect home-use bleaching gel.

It has been observed that remineralising agents largely compensate for the decrease in microhardness that occurs after bleaching [40].

To observe the effect of application duration on the morphological structure of enamel exposed to bleaching treatments, Vilhena et al. applied a home-use bleaching agent containing 10% CP for 14, 21, and 28 days. They concluded that the application duration did not have a significant effect on surface roughness; however, the changes in microhardness values were inversely proportional to the application durations [41]. Pimenta-Dutra et al. applied home-use bleaching agents containing 10% and 16% CP for 14 days (8 h a day) to investigate the effect of gel concentration and application duration on the enamel surface. It was concluded that there was a significant difference between the groups in terms of surface roughness and that surface roughness increased with CP concentration [42]. In our study, bleaching gels containing CP were applied for 4 h a day and bleaching gels containing HP were applied for 1 h a day, both for 14 days; however, no significant negative effects regarding microhardness or surface roughness values were observed.

Monterubbianesi et al. investigated whether the addition of nanohydroxyapatite could be a safe solution for teeth bleaching, without altering the microstructure or microhardness of the teeth. They compared an at-home bleaching gel containing 6% HP with the same gel enriched with nanohydroxyapatite. Although the home-use bleaching gel containing 6% HP produced slightly lower average microhardness values than the same gel enriched with nanohydroxyapatite, the difference between the two groups was not significant. However, it has been reported that nanohydroxyapatite largely preserves enamel surface morphology and compensates for increased surface roughness [43].

Arnaud et al. applied chitosan to tooth enamel during remineralisation after demineralisation and reported that the lowest phosphate loss was in the chitosan treatment group. In addition, the groups were evaluated in terms of microhardness, and it was reported that the most favourable results were also observed in the chitosan group [13]. In a study evaluating the effect on enamel microhardness of an xperimental 6% HP bleaching gel enriched with chitosan, Ozcetin et al. concluded that chitosan prevented a decrease in microhardness without affecting the bleaching effectiveness of the gel [32].

The artificial saliva used in this study (Ca^2+^, PO_4_^3−^, pH 5.5) represents a mildly acidic environment rather than a neutral one, and therefore mimics mildly demineralising conditions. Enamel begins to demineralise at pH values below approximately 5.5, whereas physiological saliva (pH 6.5–7.2) supports remineralisation due to its buffering capacity and mineral content [44,45]. This condition was chosen to evaluate how the tested formulations perform under slight demineralization stress, which may influence enamel response during bleaching. This explanation has been added to justify our experimental setup. Although the artificial saliva contained calcium and phosphate ions, the acidic pH may have limited optimal mineral recovery. These conditions could have influenced the degree of microhardness improvement observed in the study, and we acknowledge this as a methodological limitation.

In an in vitro study investigating the effect of theobromine on surface microhardness, enamel sections taken from premolars were divided into four groups and treated with distilled water, 100 mg/L, 500 mg/L, or 1000 mg/L theobromine solutions. The treatments were applied for 15–80 min. After the microhardness test, an increase was observed in the microhardness of the samples treated with the theobromine solutions, and the highest increase was reported in the group treated with the 1000 mg/L theobromine solution [46].

Another study by Taneja et al. compared theobromine and commercially available remineralising agents. They reported no significant difference following SEM-EDX analysis and stated that theobromine could be used as an alternative remineralising agent [47]. Farhad et al. initially investigated the effectiveness of theobromine on the remineralisation of enamel caries lesions by comparing it with 0.05% sodium fluoride solution and reported that the remineralisation effect of theobromine was significantly higher than that of sodium fluoride [48]. In contrast, Danaswari et al. reported that theobromine prevented a decrease in surface microhardness after bleaching treatment; they determined that theobromine reduces the increase in microhardness values [49].

Chitosan has been widely documented to exhibit antibacterial, antibiofilm, and anti-demineralising effects, reducing plaque accumulation and supporting enamel integrity through its cationic binding ability and modulation of mineral ion exchange [10,11,12]. Similarly, theobromine has been reported to increase enamel microhardness, reduce demineralisation, and promote crystal growth in the early stages of enamel lesions, suggesting cariostatic potential that may complement bleaching procedures [15].

The absence of detrimental changes in microhardness and roughness in the ECP group is consistent with similar previously reported protective and remineralising properties. This suggests that the synergistic presence of chitosan and theobromine may help counteract potential mineral loss associated with peroxide-based bleaching, thereby contributing to safer formulations.

### 2.6. Colour Evaluation

High positive WI_D_ values indicate that the whiteness values of the samples are high, while low values indicate that the whiteness levels of the samples are low [50]. In our study, WI_D_ values measured during and at the end of the bleaching treatments increased significantly in all groups, and the highest increase was observed following treatment with FGM 470 (Group 4). In our study, colour changes were evaluated according to CIEL*a*b* and ΔWI_D_ formulations, and effective changes in colour and whiteness were observed in all groups. A comparison of the ΔE_Lab1_ and ΔWI_D1_ values revealed a difference between the values measured before the bleaching treatment and on the seventh day of bleaching. We made separate measurements on the seventh day of bleaching—that is, half of the total bleaching time—and at the end of the treatment to determine between which days the increases in ΔE_Lab_ and ΔWI_D_ values were highest. A noticeable change occurred in all groups within the first 7 days, with ΔE_Lab1_ greater than 3.2 and ΔWI_D1_ greater than 2.60.

Colour changes occurred in all groups during the first 7 days of treatment, and there was a significant difference between ΔE_Lab1_ and ΔE_Lab2_, but not between ΔE_Lab2_ and ΔE_Lab3_ values and the ΔE_Lab4_ value. In the first 7 days of treatment, the change in whiteness was high in all groups, and there was a significant difference in ∆WI_D1_ and ∆WI_D2_ but no significant difference in the ∆WI_D2_ and ∆WI_D3_ and ∆WI_D4_ values.

Lilaj et al. compared the effectiveness of home-use bleaching gels containing 10% and 16% CP and home-use bleaching gels containing 6% HP and found no significant difference in the ∆WI_D_ values between treatment groups [51]. Costacurta et al. compared the efficacy of bleaching agents containing 10% and 22% CP and concluded that the ∆E and ∆WI_D_ values following treatment with either gel were similar [52].

Although there was a positive correlation between the ΔE_Lab_ and ∆WI_D_ values in terms of the evaluation of colour change in our study, the results were not compatible with each other. We believe that this was observed because the WI_D_ values are affected by each of the L*, a*, and b* values at different rates, and the a* value, which has the highest coefficient in the WI_D_ formulation, was significantly different between the groups. Consistent with our findings, Bernardi et al. evaluated the effects of bleaching agents containing 4% and 10% HP on colour change and a whiteness index and found that the ∆WI_D_ values were higher for the gel with a 10% HP content. They emphasised that the most important index for evaluating bleaching effectiveness is WI_D_ [53]. Likewise, in a study in which Pini et al. aimed to evaluate the effects on tooth properties and bleaching effectiveness of adding chitosan to an in-office bleaching gel containing 35% HP, the authors reported no significant difference between the gels with and without chitosan in terms of ΔE_00_ and ΔWI_D_ values [31]. However, the groups with the highest ΔE_00_ and ΔWI_D_ values supported our study’s results. Ozcetin et al. compared the bleaching efficacy of an experimental 6% HP bleaching gel enriched with chitosan and bleaching gels containing 25% and 40% HP. They found no significant differences between the groups. In another study, Ozcetin et al. evaluated the bleaching efficacy and 3-month follow-up results of an experimental bleaching gel containing 6% HP and enriched with chitosan, and reported that the gel provided effective bleaching and that there was no significant return in tooth colour after 3 months [54].

No studies in the literature have reported the use of chitosan and theobromine, which are used in bleaching applications in many areas, including dentistry and medicine, and our study is the first to evaluate this subject.

Within the limitations of this study, experimental bleaching gels enriched with chitosan and theobromine provided us with new alternatives to existing bleaching treatments and an opportunity to avoid the unwanted side effects of these treatments.

In our study, we believed that theobromine inhibited the effects, in terms of increasing the surface roughness and reducing the microhardness of superficial enamel layers, of the HP and CP added to our experimental gels. Studies in the literature suggest that theobromine prevents a decrease in microhardness and reduces surface roughness via its remineralisation properties, and the results of our study are consistent with this data.

The findings of the present study demonstrate that the incorporation of chitosan and theobromine into a bleaching gel (ECP group) did not produce any adverse effects regarding enamel surface roughness, microhardness, or colour stability. On the contrary, the colour change (ΔE_Lab) and whitening index (ΔWID) observed in the ECP group were comparable to those of conventional bleaching formulations, indicating that the addition of these bioactive components does not compromise whitening efficacy.

We believe that chitosan, which was used as an alternative to thickeners and carrier synthetic polymers in our experimental gels, prevented the negative effects of bleaching treatments on the enamel surface due to its bioadhesive and remineralising properties. Furthermore, we believe that chitosan slows the release of HP and CP from gels to the external environment and increases bleaching efficiency at low concentrations.

A preliminary version of this manuscript was published as a preprint on Research Square [55].

This study has several limitations. First, it was conducted in vitro and therefore cannot fully simulate the biological conditions of the oral environment. The use of artificial saliva at pH 5.5 represents a mildly acidic medium that does not reflect physiological salivary pH; this may have introduced an additional demineralisation challenge that differs from typical clinical conditions. Furthermore, enamel specimens were stored ex vivo, and potential dehydration effects, despite controlled handling, may have influenced the surface properties measured. In addition, maximum surface polishing was not performed before roughness assessments, and the low load and short application duration used for microhardness testing may have affected measurement sensitivity. Another limitation is that the biocompatibility of the chitosan- and theobromine-containing bleaching gels was not evaluated, as the study focused only on enamel surface outcomes. Although previous research suggests that both components individually demonstrate favourable biocompatibility, future in vitro and in vivo studies are necessary to confirm the safety and effectiveness of these experimental formulations under clinically relevant conditions. Natural saliva flow, enzymatic activity, pH fluctuations, and mechanical factors such as chewing or brushing cannot be adequately reproduced in laboratory settings. Patient-related factors, including oral hygiene, diet, and compliance, may also influence enamel responses to bleaching agents. Moreover, differences between static artificial saliva immersion and dynamic clinical bleaching dynamics limit the direct generalisation of our findings. Therefore, further in vivo or clinically simulated studies are required to validate these results.

## 3. Conclusions

After 14 days of follow-up, none of the bleaching or bioactive gel formulations caused significant changes in enamel microhardness or surface roughness, indicating that the treatments did not adversely affect enamel integrity. The ECP group (Group 1), containing chitosan and theobromine, showed the greatest improvements in terms of colour change and whitening index, suggesting enhanced whitening efficiency without the enamel surface properties being compromised. These findings support previous evidence that chitosan and theobromine may provide protective and remineralising benefits, highlighting their potential use as bioactive components in safer bleaching systems. However, as this was an in vitro study, further in vitro and in vivo research—particularly long-term and clinical studies—is needed to confirm the antibacterial effects, remineralisation potential, and overall clinical performance of these formulations.

## 4. Materials and Methods

Ethics committee approval (number 2022/310) was obtained from the Gaziantep University Clinical Research Ethics Committee for this study. In the calculation of colour change values, the number of samples for each group was determined at a 0.05 significance level, the power of the test was 0.80 (1 − β), and the effect size was 12 for 0.42. In the calculation of the change in microhardness, the number of samples for each group was determined at a 0.05 significance level, the strength of the test was 0.80 (1 − β), and the effect size was determined to be 12 for 0.35. In the calculation of surface roughness, the number of samples for each group was determined at a 0.05 significance level, the strength of the test was 0.90 (1 − β), and the effect size was determined to be 12 for 0.45.

**Figure 1:**
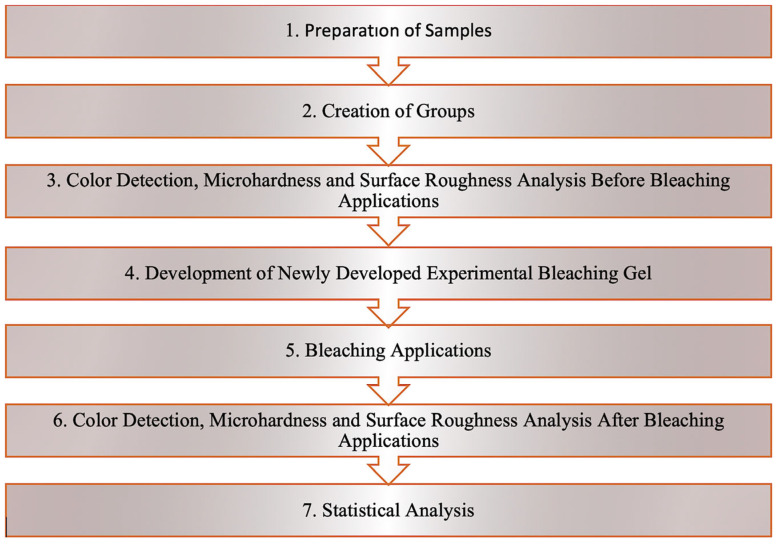
Study protocol.

The study protocol is shown in Figure 1.

### 4.1. Preparation of the Teeth Used in the Study and Formation of the Groups

We used 144 human upper central incisors in this study. All samples were standardised in terms of enamel-dentin thickness using a CIBT device (ProMax 3D; Planmeca, Helsinki, 105 Finland). The 144 teeth were divided into four groups according to the bleaching gel treatment (n = 36). The groups were then divided into three subgroups for measurement of colour (n = 12), surface roughness (n = 12), and microhardness (n = 12). The names and descriptions of the four experimental groups:

Group1: ECP (experimental gel containing chitosan + theobromine with Carbamide Peroxide)

Group 2: EHP (experimental gel without bioactive agents with Hydrogene Peroxide)

Group 3: BIO (BioWhiten-ProHome gel)

Group 4: FGM (FGM-Whiteness Perfect gel)

### 4.2. Preparation of Samples for Microhardness and Surface Roughness Studies

The roots of 96 upper central incisors, which were used to measure microhardness and surface roughness, were separated from the crown part with diamond discs under water cooling to be 2 mm apical to the cementum–enamel border. Each root was then embedded separately in pink autopolymerising acrylic resin (Imicryl SC, Imicryl Dental, Konya, Turkey) so that the labial surface of the tooth remained outside and parallel to the ground plane.

### 4.3. Preparation of Samples for Colour Analysis

Forty-eight upper middle incisors, which were to be used for colour analysis, were embedded in a silicone measuring material (Heavy-Body, Optosil; Heraeus Kulzer, Hanau, Germany) in groups of six, 1 mm above the enamel–cement border, with their crowns exposed. We applied 1 mm of permissible silicone measuring agent (Light-Body Optosil; Heraeus Kulzer, Hanau, Germany) along the enamel–cement margin of the teeth to prevent gel spread to the surrounding gingiva during plaque use.

### 4.4. Preparation of Devices for Home-Use Bleaching

Before the bleaching agent was applied, the acrylic blocks used for measuring microhardness and surface roughness and the silicone blocks used for colour measurement were measured using an alginate measuring agent (Cavex Color Change; Cavex Holland BV, Haarlem, The Netherlands) with the appropriate spoon; the models were obtained from hard plaster (IMISTONE Dental Hard Stone, Imicryl, Konya, Turkey). To create reservoir areas for the bleaching gels on the plaster models, 1 mm thick resin blocks (LC Block-Out Resin, Ultradent Products, Inc., South Jordan, UT, USA) were placed on the buccal surface of all teeth, 1.5 mm away from the determined gingival border, and a soft, 0.035-inch (0.9 mm)-thick bleaching plate was printed using a vacuum machine (Vacuum Forming Machine, Keystone İndustries, Myerstown, PA, USA). Bleaching applications were performed using this plaque in a metal bathtub at 37 °C with a sterilisation bag and a small amount of artificial saliva on the base.

### 4.5. Preparation of Artificial Saliva Solution

An artificial saliva solution was created at the Department of Biochemistry of Gaziantep University, where the teeth were stored when the procedure was not being performed. A total of 0.3282 g of calcium nitrate 4-hydrate (Ca[NO_3_]_2_ 4H_2_O) and 1.3613 g potassium hydrophosphate (KH_2_PO_4_) were dissolved separately in 600 mL distilled water. These two solutions were collected in a single container, which was filled to 2000 mL with distilled water. The solvent was adjusted to neutral by adding 0.1 M potassium acetate (CH_3_COOK), and a 4 mL artificial saliva solution containing 0.002 M Ca(NO_3_)_2_ and 0.01 M KH_2_PO_4_ with a pH of 5.5 was thus prepared. The artificial saliva solution in which the teeth were stored was changed daily.

### 4.6. Preparation of Experimental Bleaching Gel

Using a precision balance (Sartorius, 214), 1% acetic acid and 2 g chitosan (Sigma Aldrich Chemical, St. Louis, MO, USA) were weighed, added to 100 mL of water, and dissolved in 1% acetic acid using a magnetic stirrer (FAITHFUL SH-2). Subsequently, 0.05 g of theobromine (Sigma Aldrich Chemical, St. Louis, MO, USA) was added. The prepared solution was mixed with 30% HP (Sigma Aldrich Chemical, St. Louis, MO, USA) at the determined ratios to obtain 6% HP gel; 16% CP gel was obtained by mixing 97% CP (Sigma Aldrich Chemical, St. Louis, MO, USA) at the determined ratios.

### 4.7. Vickers Microhardness Measurement

A Vickers microhardness tester (LHV-1D, URNDT Co., Ltd., Beijing, China) was used to measure the microhardness values of the samples prepared for bleaching applications. A 200 g force was applied to the enamel surface for 10 s, and pyramid-shaped marks were produced on the surface of the teeth. The two parallel lines in the device’s microscope were adjusted to be tangent to the corners of the pyramidal indentation, and the microhardness was then calculated. The hardness was measured in inverse proportion to the size of the indentation formed on the tooth surface. Measurements were made at three different points, and the average of these values was recorded as the Vickers hardness number (VHN). All tooth samples were divided into groups, and no significant differences in microhardness values were measured between groups before bleaching.

### 4.8. Surface Roughness Measurement Using a Profilometer

Surface roughness testing was performed on the prepared samples using a contact-type profilometer (SurftestSJ-400, Mitutoyo, Kawasaki, Japan) prior to the bleaching treatment. The device was standardised with a reading length of 1.25 mm, a cutting length of 0.25 mm, and a speed of 0.05 mm/s.

Holes with a diameter of 6 mm were drilled into 0.040-inch (1.5 mm)-thick hard plates prepared to standardise the measurement of surface roughness in an area corresponding to the middle triad, parallel to the buccal surface of the teeth. The first measurement was made along a line from the incisal part to the cervical part in the middle triad of the teeth. Following this measurement, the second and third measurements were made in such a way that they converged at the centre counterclockwise and along the directions moving at an average angle of 60°; the average of the three measurements was taken in Ra.

All teeth were divided into groups, with no significant differences in surface roughness values measured between groups prior to bleaching.

### 4.9. Colour Measurement Using a Spectrophotometer

Colour measurements were made using a VITA Easyshade Advance 4.0 (Zahnfabrik, H. Rauter GmbH & Co., Bad Sackingen, Germany) spectrophotometer before the application of bleaching treatment to the prepared samples. Colour measurements were performed under D65 daylight on a neutral grey background, touching the centre of the middle third of the tooth surface. Holes with a diameter of 6 mm were drilled into 0.040-inch (1.5 mm)-thick hard plates prepared to ensure standardisation during the colour measurements. Each measurement was made three times from these holes, and the average of L*, a*, and b* values was calculated. All teeth that did not differ significantly regarding L*, a*, and b* values measured before bleaching were divided into groups.

Based on the results obtained from these measurements, the colour difference (∆E_Lab_) and the whiteness index (∆WI_D_) were calculated, and the results were recorded.

The colour change values were evaluated using the CIELAB system, which is preferred in many studies and provides quantitative results. A value of ∆E ≥ 3.3 was accepted as the threshold value for the detection of a colour change in clinical observation. A detectability threshold of IC WI_D_ = 0.72 and an acceptability threshold of IC WI_D_ = 2.60 were adopted.

Between the bleaching sessions, the bleaching gel on the teeth was washed off with distilled water for 30 s, and the teeth were decontaminated with a soft toothbrush for 20 s. The apparatus was cleaned under running water. Between sessions, the teeth were stored in an artificial saliva solution in an oven at 37 °C.

### 4.10. Statistical Analysis

The suitability of numerical variables to a normal distribution was evaluated using the Shapiro–Wilk test, and the homogeneity of variances was assessed with Levene’s test. When both assumptions were satisfied, parametric tests were applied: ANOVA followed by LSD post hoc tests for intergroup comparisons, and the paired *t*-test for within-group comparisons. When the assumptions of normality or homoscedasticity were violated, non-parametric alternatives were used. Specifically, the Kruskal–Wallis test followed by Dunn’s multiple comparison test was applied for intergroup analyses, and the Wilcoxon signed-rank test was used for within-group comparisons. Correlation analyses were performed using Spearman’s rho test. Correlation strength was classified as high for r > 0.60, moderate for r = 0.30–0.60, and low for r < 0.30. A *p*-value < 0.05 was considered statistically significant. All statistical analyses were conducted using SPSS Statistics 22.0 software (IBM Corp., Armonk, NY, USA).

**Figure 2:**
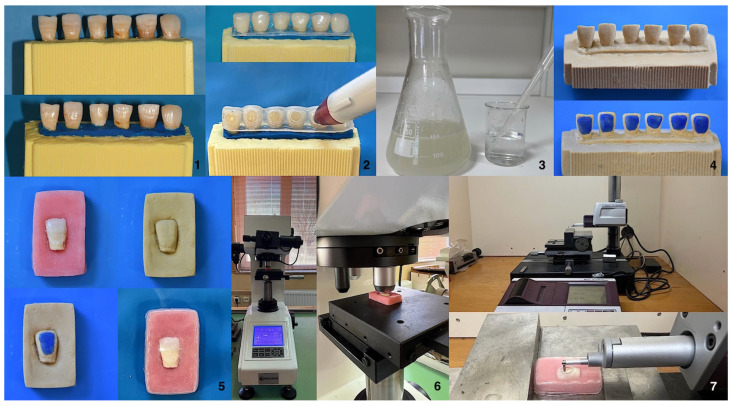
All study protocols are presented. (**1**) Selection of the teeth; (**2**) Color detection; (**3**) Preparation of bleaching gel; (**4**,**5**) Preparation of the teeth for bleaching application; (**6**) Profilometer and surface roughness measurement; (**7**) Microhardness tester and microhardness measurement.

All study protocols are presented schematically in a step-by-step manner in Figure 2.

## Figures and Tables

**Table 1:** Mean Vickers microhardness values before and after bleaching (VHN ± sd).

	VHN_1_ ± sd	VHN_2_ ± sd	VHN_3_ ± sd
ECP	97.55 ± 4.29 ^Aa^	98.92 ± 3.03 ^Aa^	98.63 ± 1.92 ^Aa^
EHP	98.78 ± 1.14 ^Aa^	98.85 ± 2.45 ^Aa^	98.07 ± 2.16 ^Aa^
BIO	97.78 ± 2.93 ^Aa^	97.80 ± 3.20 ^Aa^	97.33 ± 2.97 ^Aa^
FGM	97.63 ± 4.34 ^Aa^	97.77 ± 4.81 ^Aa^	97.30 ± 4.56 ^Aa^
*P*	0.802	0.755	0.675

^Aa^—Different letters in columns and rows indicate statistically significant differences. Lowercase letters represent row differences, uppercase column differences (*p* > 0.05).

**Table 2:** Mean surface roughness values before and after bleaching (Ra ± sd).

	Ra_1_ ± sd	Ra_2_ ± sd	Ra_3_ ± sd
ECP	0.848 ± 0.478 ^Aa^	0.673 ± 0.503 ^Ab^	0.670 ± 0.530 ^Ab^
EHP	0,.799 ± 389 ^Aa^	0.637 ± 273 ^Ab^	0.615 ± 0.278 ^Ab^
BIO	0.820 ± 211 ^Aa^	0.986 ± 0.210 ^Ba^	0.971 ± 0.209 ^Ba^
FGM	0.768 ± 0.153 ^Aa^	0.705 ± 0.112 ^Ab^	0.701 ± 0.101 ^Ab^
*P*	0.946	0.033	0.044

^Aa^, ^Ab^ and ^Ba^—Different letters in columns and rows indicate statistically significant differences. Lowercase letters represent row differences, uppercase column differences (*p* > 0.05).

**Table 3:** Mean ΔE_Lab_ and standard deviation values of all groups (Mean ± sd).

	ECP	EHP	BIO	FGM	*P*
ΔE_Lab1_	8.45 ± 3.38 ^Aa^	6,17 ± 3.91 ^Aa^	6.88 ± 2.87 ^A^	8.52 ± 4.05 ^Aa^	0.297
ΔE_Lab2_	11.20 ± 4.43 ^Ba^	9.69 ± 3.33 ^Ba^	9.19 ± 3.22 ^B^	11.32 ± 4.67 ^Ba^	0.463
ΔE_Lab3_	12.14 ± 5.66 ^Ba^	9.31 ± 3.15 ^Ba^	9.01 ± 3.84 ^B^	11.86 ± 3.87 ^Ba^	0.157
ΔE_Lab4_	11.76 ± 5.29 ^Ba^	9.00 ± 2.78 ^Ba^	8.59 ± 3.90 ^B^	10.53 ± 3.62 ^Ba^	0.207

^A^, ^Aa^, ^B^ and ^Ba^—The letters in the rows and columns indicate a statistically significant difference. Capital letters indicate statistical difference within the same column; lowercase letters indicate statistical difference within the same row (*p* < 0.05).

**Table 4:** Mean ΔWI_D_ and standard deviation values of all groups (mean ± sd).

	ECP	EHP	BIO	FGM	*p*
ΔWID1	9,35 ± 8.03 ^Aa^	7,62 ± 7.05 ^Aa^	8,60 ± 3.55 ^Aa^	10,83 ± 7.39 ^Aa^	0.214
ΔWID2	14.91 ± 10.47 ^Ba^	12.98 ± 3.84 ^Ba^	12.13 ± 4.47 ^Ba^	16.29 ± 9.47 ^Ba^	0.562
ΔWID3	15.42 ± 11.24 ^Ba^	12.56 ± 3.66 ^Ba^	12.29 ± 5.86 ^Ba^	16.33 ± 7.88 ^Ba^	0.258
ΔWID4	15.23 ± 11.92 ^Ba^	12.69 ± 4.10 ^Ba^	11.82 ± 5.73 ^Ba^	15.13 ± 8.12 ^Ba^	0.601

^Aa^ and ^Ba^—Letters in rows and columns indicate a statistically significant difference. Capital letters represent statistical difference within the same column; lowercase letters are within the same row (*p* < 0.05).

**Table 5:** Correlation values between ΔE_Lab_-ΔWI_D_ formulas (r).

	ΔE_Lab1_-ΔWI_D1_	ΔE_Lab2_-ΔWI_D2_	ΔE_Lab3_-ΔWI_D3_	ΔE_Lab4_-ΔWI_D4_	*P*
ECP	0.73 *	0.84 *	0.89 *	0.92 *	<0.05
EHP	0.71 *	0.90 *	0.80 *	0.77 *	
BIO	0.84 *	0.81 *	0.87 *	0.88 *	
FGM	0.83 *	0.76 *	0.71 *	0.81 *	

* Significant at *p* < 0.05, high at r > 0.6.

## Data Availability

The original contributions presented in this study are included in the article. Further inquiries can be directed to the corresponding author.
